# Synthesis of Pyrochlore Oxides Containing Ir and Ru for Efficient Oxygen Evolution Reaction

**DOI:** 10.3390/ma15176107

**Published:** 2022-09-02

**Authors:** Aika Matsumoto, Ze-Xing Cai, Takeshi Fujita

**Affiliations:** School of Environmental Science and Engineering, Kochi University of Technology, 185 Miyanokuchi, Tosayamada, Kami, Kochi 782-8502, Japan

**Keywords:** pyrochlore oxide, water splitting, iridium oxide, ruthenium oxide, electrocatalysis

## Abstract

A versatile synthesis method for pyrochlore oxides containing Ir and Ru with lanthanides Pr, Nd, Eu, Gd, Tb, and Ho is herein presented. Based on the systematic synthesis and Rietveld refinement results, the lattice constants were tunable depending on the ionic radius of the lanthanide used. Subsequently, Pr-based pyrochlore oxides containing Ru and Ir in different ratios were fabricated for the oxygen evolution reaction (OER) in alkaline media, and the OER activity of these catalysts increased when the content of Ir and Ru was the same. Thus, the co-substitution of Ir and Ru in pyrochlore oxides is a novel synthesis strategy for electrocatalysts, which provides great potential for the fabrication of other pyrochlore oxides with various architectures and compositions for application in electrocatalysis.

## 1. Introduction

Global energy consumption increases by 2.5% (in oil equivalent) annually. To realize a sustainable society, water-splitting technology using renewable energy is attractive for the production of hydrogen as an alternative to oil [1,2,3]. Electrochemical water splitting involves two reactions: the hydrogen evolution reaction (HER) and the oxygen evolution reaction (OER). Based on systematic studies, the relationship between surface-molecule interactions and performance (known as the “volcano plot”) is a useful indicator to predict activity [4,5]. Considering the active materials from the volcano plot of the OER, IrO_2_ and RuO_2_ are the active oxides. Moreover, multiple studies have reported the improved OER activities of Ir_x_Ru_1−x_O_y_ under full-cell testing conditions [6,7,8,9,10,11,12,13,14]. The electrochemical stability related to Ir/Ru dissolution is an essential mechanism for further improvement and optimization of the process [15].

Recently, pyrochlore-type oxides (A_2_B_2_O_7_) have been reported as a promising new class of OER catalysts [16]. These oxides possess a crystal framework that is capable of simultaneously accommodating Ir and Ru at the B sites, which strengthens the interaction between local RuO_6_ and IrO_6_ via shared oxygen and O–A–O bonds [17]. Furthermore, recent studies have reported the elemental feasibility of forming high-entropy pyrochore oxides by heavy doping of lanthanides on the A site and transition metals on the B site [18,19,20,21]. In this study, A_2_Ru_2_O_7_ and A_2_Ir_2_O_7_, where A is a lanthanide (Pr, Nd, Eu, Gd, Tb, and Ho), are synthesized systematically to confirm the feasibility of synthesis and obtain crystal information. Subsequently, Pr_2_(Ru_x_Ir_1−x_)_2_O_7_ with different x values is synthesized for application as OER catalysts because Pr-based pyrochore oxides exhibit good conductivity and are suitable as an electrode catalyst among the lanthanides [22]. Pr_2_(Ru_0.5_Ir_0.5_)_2_O_7_ with an identical Ir and Ru same atomic percentage and uniform element distribution exhibited good OER activity and stability compared with the benchmark IrO_2_ catalyst. Pyrochore oxide with Ir and Ru has controllable and flexible compositions, which is important for its potential application in various fields.

## 2. Materials and Methods

### 2.1. Chemicals

Ruthenium (III) nitrosylnitrate, iridium acetate, praseodymium (III) acetate, neodymium acetate monohydrate, europium (III) acetate n-hydrate, gadolinium (III) acetate tetrahydrate, terbium (III) acetate tetrahydrate, holimium (III) acetate monohydrate (99.9%), L-aspartic acid (L-AA), hydrochloric acid, iridium (IV) oxide, 2-propanol, a 5 wt% Nafion dispersion solution, potassium hydroxide, and potassium chloride were purchased from FUJIFILM Wako Pure Chemical Corporation. All the chemicals were used without further purification. Ultrapure deionized water, which was prepared using a Millipore system (Milli-Q), was used in the measurements.

### 2.2. Synthesis of A_2_Ru_2_O_7_

Using Pr_2_Ru_2_O_7_ as an example, a precursor was prepared using an amino acid-facilitated method [23]. Subsequently, (CH_3_COO)_3_Pr·nH_2_O (107.6 mg, 0.315 mmol), RuN_4_O_10_ (100 mg, 0.315 mmol), and L-AA (125.8 mg, 0.945 mmol) were dissolved in 50 mL of deionized water. The resulting solution was stirred at 60 °C for 3 h and then heated in an oven at 150 °C for 12 h to obtain the solid precursor. The solid was then ground using a mortar and pestle (1–2 min) to obtain a fine powder. Subsequently, the powdered sample was transferred to an alumina boat, calcined in air at 1050 °C for 3 h, and cooled to room temperature. For the other solid-solution perovskites, the amount of A sites was changed, but the other synthesis conditions were not. The samples were obtained using (CH_3_COO)_3_Nd·H_2_O (106.9 mg, 0.315 mmol), (CH_3_COO)_3_ Eu·nH_2_O (126.3 mg, 0.315 mmol), (CH_3_COO)_3_Gd·4H_2_O (128.0 mg, 0.315 mmol), (CH_3_COO)_3_Tb·4H_2_O (128.6 mg, 0.315 mmol), and (CH_3_COO)_3_Ho·H_2_O (107.9 mg, 0.315 mmol) in the synthesis mixtures.

### 2.3. Synthesis of A_2_Ir_2_O_7_

Similarly, (CH_3_COO)_3_Pr·nH_2_O (96.6 mg, 0.283 mmol), Ir(OCOCH_3_)_n_ (100 mg, 0.283 mmol), and L-AA (113 mg, 0.849 mmol) were dissolved in 50 mL of deionized water. The resulting solution was stirred at 60 °C for 3 h and heated in an oven at 150 °C for 12 h to obtain the solid precursor. Subsequently, the solid was ground using a mortar and pestle (1–2 min) to obtain a fine powder. The powdered sample was then transferred to an alumina boat, calcined in air at 1050 °C for 3 h, and cooled to room temperature. For the other solid-solution perovskites, the samples were obtained using (CH_3_COO)_3_Nd·H_2_O (96.0 mg, 0.283 mmol), (CH_3_COO)_3_ Eu·nH_2_O (113.5 mg, 0.283 mmol), (CH_3_COO)_3_Gd·4H_2_O (115 mg, 0.283 mmol), (CH_3_COO)_3_Tb·4H_2_O (115.5 mg, 0.283 mmol), and (CH_3_COO)_3_Ho·H_2_O (96.8 mg, 0.283 mmol) in the synthesis mixtures.

### 2.4. Synthesis of Pr_2_(Ru_x_Ir_1−x_)_2_O_7_

The synthesis of Pr_2_(Ru_0.5_Ir_0.5_)_2_O_7_ (x = 0.5) is presented as an example. (CH_3_COO)_3_Pr·nH_2_O (150 mg, 0.440 mmol), RuN_4_O_10_ (69.6 mg, 0.220 mmol), Ir(OCOCH_3_)_n_ (77.8 mg, 0.220 mmol), and L-AA (175.4 mg, 1.317 mmol) were dissolved in 50 mL of deionized water. The resulting solution was stirred at 60 °C for 3 h and heated in an oven at 150 °C for 12 h to obtain the solid precursor. Subsequently, the solid was ground using a mortar and pestle (1–2 min) to obtain a fine powder. The powdered sample was then transferred to an alumina boat, calcined in air at 1050 °C for 3 h, and cooled to room temperature. For the other solid-solution pyrochlores, Pr_2_(Ru_x_Ir_1−x_)_2_O_7_ with different molar ratios of Ru and Ir were used.

### 2.5. Electrochemical Characterization

Electrochemical experiments were conducted using an IviumStat electrochemical workstation (IVIUM Technologies B.V., Eindhoven, The Netherlands) with a typical three-electrode cell setup. Pt wire and Ag/AgCl (in saturated KCl) were used as the counter and reference electrodes, respectively. Glassy carbon (5 mm in diameter) was prepared using a catalyst ink suspension and served as the working electrode. Catalyst ink suspensions were prepared using 5 mg of the pyrochlore catalyst, 100 μL of deionized water, 900 μL of isopropyl alcohol, and 40 µL of 5 wt% Nafion solution. The catalyst ink suspensions were sonicated for 30 min before depositing 5 µL onto a polished glassy carbon disk electrode (0.19625 cm^2^) to obtain a total catalyst loading of ~127.4 μg/cm^2^. The electrolyte (1.0 M KOH) was prepared using potassium hydroxide and saturated with synthetic air for all measurements.

Linear sweep voltammetry (LSV) was performed at a potential scan rate of 5 mV/s for all electrolytes. Tafel plots were obtained from the LSV curve using the formula *η* = *b* × log *j* + *a*, where *η* is the overpotential, *j* is the current density, and *b* is the Tafel slope. For the stability test by chronoamperometry, a carbon sheet with a catalyst loading of 0.5 mg/cm^2^ was used as the working electrode for improved adhesion compared with the flat carbon electrode. 

### 2.6. Structural Characterization

The X-ray diffraction (XRD) patterns of the samples were obtained using a Rigaku RINT 2000 X-ray diffractometer equipped with a monochromatic Cu Kα radiation unit (40 kV, 40 mA). The microstructure of the samples was analyzed by scanning electron microscopy (SEM) using a Hitachi S-8020 scanning electron microscope operated at an accelerating voltage of 10 kV. The microstructure of the samples was further characterized by transmission electron microscopy (TEM) using a transmission electron microscope (JEM-ARM200F “NEO ARM,” JEOL) equipped with aberration correctors for the image- and probe-forming lens systems (CEOS GmbH) and an energy-dispersive X-ray spectrometer (EDS; JED-2300T, JEOL). The TEM and scanning TEM (STEM) observations were conducted at an accelerating voltage of 200 kV.

## 3. Results and Discussion

**Figure 1:**
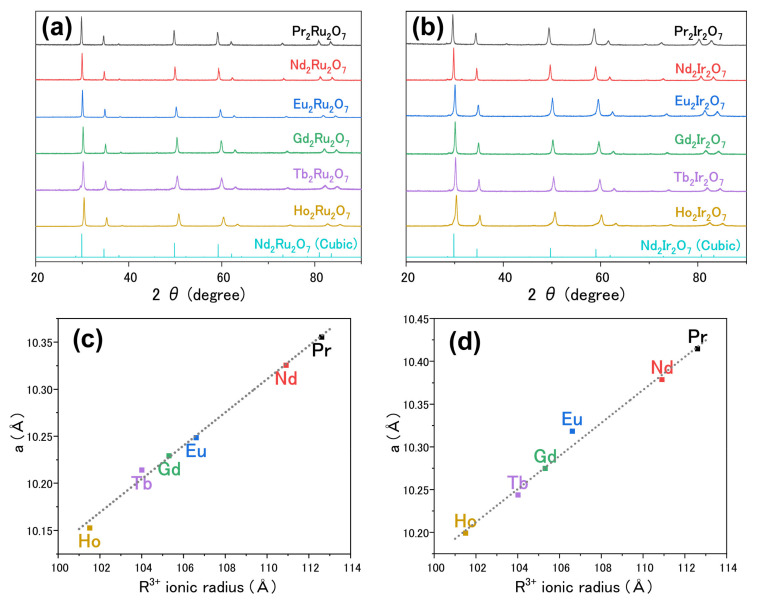
(**a**,**b**) Experimental XRD patterns of A_2_Ru_2_O_7_ and A_2_Ir_2_O_7_ where A = Pr, Nd, Eu, Gd, Tb, and Ho, using cubic Nd_2_Ru_2_O_7_ (ICSD #79327) and Nd_2_Ir_2_O_7_ (#239644) as reference samples. (**c**,**d**) Lattice constants *a* of A_2_Ru_2_O_7_ and A_2_Ir_2_O_7_ plotted against the ionic radii of R^3+^. The dotted lines serve as a visual guide.

Figure 1a,b shows a series of XRD profiles confirming the synthesis of pyrochlore-type A_2_Ru_2_O_7_ and A_2_Ir_2_O_7_ with lanthanides Pr, Nd, Eu, Gd, Tb, and Ho, using Nd_2_Ru_2_O_7_ and Nd_2_Ir_2_O_7_ as reference samples. The Rietveld refinement of the diffraction data revealed that the lattice constant *a* increased with increasing ionic radius *R*, as shown in Figure 1c,d. In pyrochlore oxides, the expansion of the lattice with *R* is usually attributed to an increase in the O–A–O bond angle via the chemical pressure effect [22].

**Figure 2:**
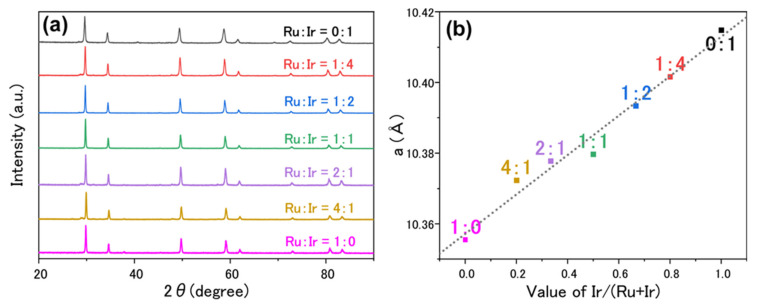
(**a**) Experimental XRD patterns of Pr_2_(Ru_x_Ir_1−x_)_2_O_7_ with different ratios of Ru and Ir. (**b**) Lattice constant *a* of Pr_2_(Ru_x_Ir_1−x_)_2_O_7_ plotted against the Ir/(Ir + Ru) molar ratio. The dotted lines serve as a visual guide.

Conductivity is an important parameter in electrocatalysis. For oxygen-evolving catalysts, high conductivity facilitates charge transfer between the catalyst–electrolyte and catalyst–support electrode interfaces. According to theoretical calculations, Pr-based pyrochore oxides exhibit the highest metallic conductivity among the lanthanides because of the disappearance of the band gap [22]. Based on the successful synthesis of Pr_2_Ru_2_O_7_ and Pr_2_Ir_2_O_7_, Pr_2_(Ru_x_Ir_1−x_)_2_O_7_ was systematically synthesized using different x values, as shown in Figure 2a. The Rietveld refinement of the diffraction data shows a linear relationship between *a* and the Ir/(Ir + Rr) molar ratio, as shown in Figure 2b.

**Figure 3:**
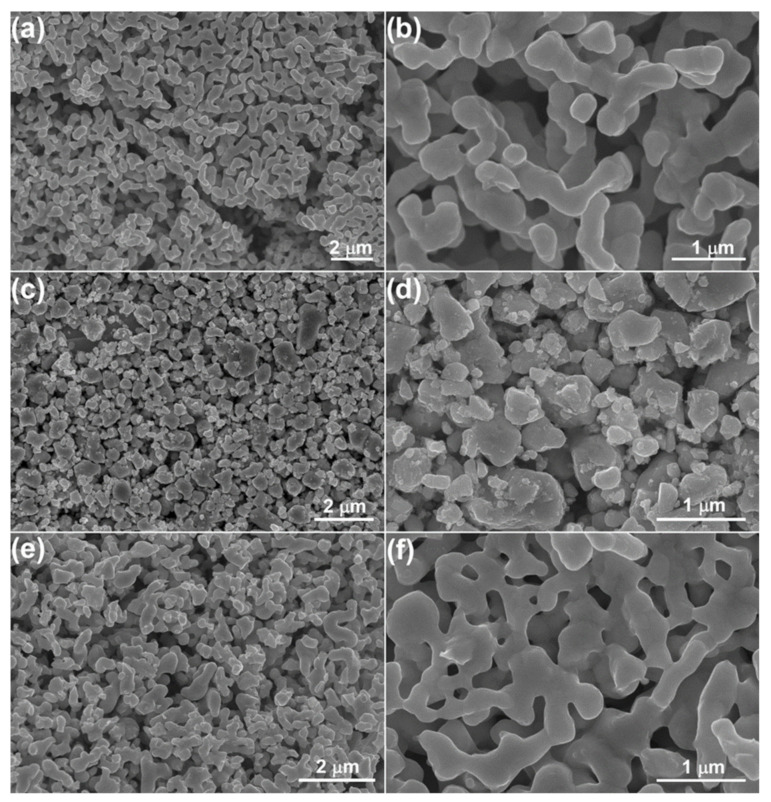
Low- and high-magnification SEM micrographs of the products. (**a**,**b**) Pr_2_Ru_2_O_7_, (**c**,**d**) Pr_2_Ir_2_O_7_, and (**e**,**f**) Pr_2_(Ru_0.5_Ir_0.5_)_2_O_7_.

**Figure 4:**
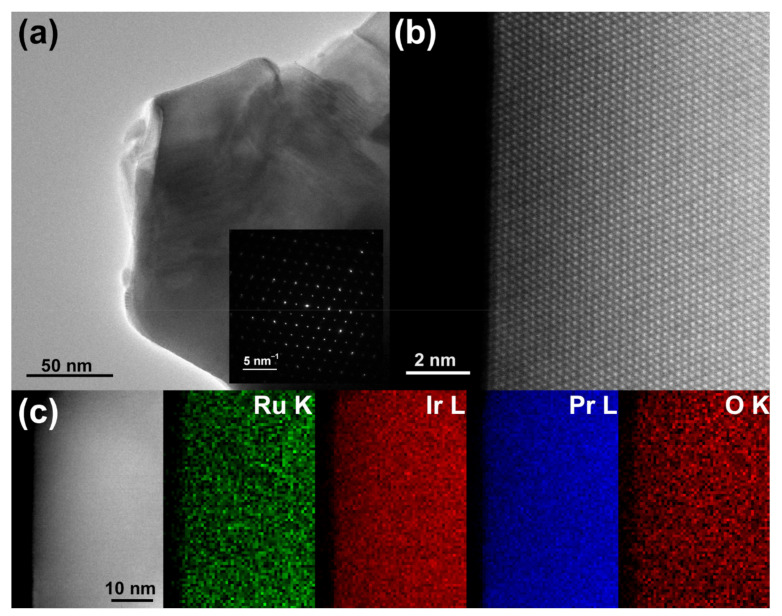
A TEM characterization of as-synthesized Pr_2_(Ru_0.5_Ir_0.5_)_2_O_7_. (**a**) Low-magnification TEM micrograph with the corresponding selected area electron diffraction (SAED) pattern along the [011] direction (inset). (**b**) HAADF-STEM image with uniform atom distribution. (**c**) EDS mappings of the Ru-K, Ir-L, Pr-L, and O-K edges.

The microstructures of Pr_2_Ru_2_O_7_, Pr_2_Ir_2_O_7_, and Pr_2_(Ru_0.5_Ir_0.5_)_2_O_7_ were characterized using SEM, as shown in Figure 3. Large particles with an average size of 400–500 nm were observed in the three samples. As expected from the high annealing temperature (1050 °C, 3 h), the nanosized character of the pyrochlore catalysts was reduced, yielding particle sizes ranging from the nano to micron scale. As shown in Figure 4a, the TEM observations along the [110] direction indicate high crystallinity owing to high-temperature annealing. As shown in Figure 4b, the high-angle annular dark field (HAADF)-STEM images indicate that no distinct local structural defects were observed. Furthermore, the atomic-scale homogeneity of each element was observed by EDS mapping shown in Figure 4c.

**Figure 5:**
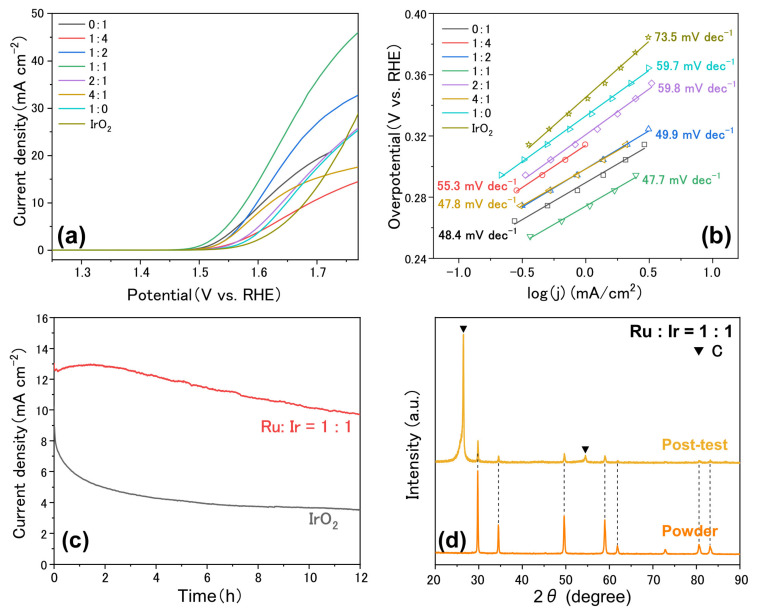
An electrocatalytic OER performance test of catalysts. (**a**) OER polarization curves of Pr_2_(Ru_x_Ir_1−x_)_2_O_7_ catalysts with different molar ratio of Ru and Ir. (**b**) Tafel plots obtained from the polarization curves in (**a**). (**c**) Chronoamperometric measurement o Pr_2_(Ru_0.5_Ir_0.5_)_2_O_7_ and commercial IrO_2_ at 10 mA/cm^2^. All the tests were performed in N_2_-saturated 1.0 M KOH solution. (**d**) Comparison of XRD profiles of post-test and as-made power samples, indicating the good phase stability. The carbon signal comes from the attached carbon paper.

The electrocatalytic OER activity of Pr_2_(Ru_x_Ir_1−x_)_2_O_7_ with different x values was investigated in a N_2_-saturated 1.0 M KOH solution at room temperature using a three-electrode electrolysis system and compared with that of commercial IrO_2_. Figure 5a shows the representative LSV curves of the electrocatalysts measured at a scan rate of 5 mV s^−1^ (with ohmic drop correction). The OER activity of the catalysts increased when the Ir and Ru contents were identical (x = 0.5). Moreover, the excellent OER performance of Pr_2_(Ru_0.5_Ir_0.5_)_2_O_7_ was further confirmed by its lower Tafel slope (47.7 mV dec^−1^) compared with the others (Figure 5b), indicating that Pr_2_(Ru_0.5_Ir_0.5_)_2_O_7_ promoted efficient OER kinetics. Comparison of some representative pyrochlore OER catalysts reported under alkaline conditions is listed in Table 1. As shown in Figure 5c, the chronopotentiometric measurements of Pr_2_(Ru_0.5_Ir_0.5_)_2_O_7_ and IrO_2_ were performed at a current density of 10 mA cm^−2^. A minor increase in the potential (versus a reversible hydrogen electrode) was observed in the *V*–*t* curves, further confirming the good stability of Pr_2_(Ru_0.5_Ir_0.5_)_2_O_7_. In contrast, the activity of IrO_2_ gradually decreased. Additionally, the structure of Pr_2_(Ru_0.5_Ir_0.5_)_2_O_7_ after the durability test was characterized by XRD (Figure 5d), which indicated that variations in the phase and lattice were negligible compared with the as-synthesized powdered samples. These results suggest that Pr_2_(Ru_0.5_Ir_0.5_)_2_O_7_ is a highly active and stable electrocatalyst for the OER in alkaline media.

Regarding to the outperformance of the Pr_2_(Ru_0.5_Ir_0.5_)_2_O_7_ catalyst, co-substitution of Ir and Ru strengthened the interaction between RuO_6_ and IrO_6_ via shared oxygen atoms and O–A–O bonds. Moreover, recent experimental and theoretical results have demonstrated that the elongated Ru–O and Ir–O bonds in the pyrochlore possesses ruthenium and iridium at a low oxidation state when their contents are similar, which contributes to a moderate binding energy for the oxygen intermediate and leads to good OER stability [17]. Further improvements are expected when the particle size is reduced to the nanometer range. Moreover, the inhibition of radical coarsening during high-temperature annealing should be investigated in future studies.

## 4. Conclusions

In this study, A_2_Ru_2_O_7_ and A_2_Ir_2_O_7_ (where A was lanthanide Pr, Nd, Eu, Gd, Tb, or Ho) were synthesized systematically to verify the synthesis route. Subsequently, Pr_2_(Ru_x_Ir_1−x_)_2_O_7_ was synthesized with different x values for application as OER catalysts. This study demonstrated that Pr_2_(Ru_0.5_Ir_0.5_)_2_O_7_ with the same Ir and Ru atomic percentages have good OER activity and stability compared with the benchmark IrO_2_ catalyst. Although the nanosized character of the pyrochlore catalysts were significantly reduced owing to high-temperature annealing during synthesis, the co-substitution of Ir and Ru in pyrochlore oxides should be applied to other material systems for achieving enhanced OER activity and stability in electrocatalytic applications.

## Figures and Tables

**Table 1:** A comparison of some representative pyrochlore OER catalysts reported under alkaline conditions.

Catalysts	Electrolyte	Overpotential (mV)	Reference
Pr_2_Ru_1_Ir_1_O_7_	1 M KOH	350@10 mA cm^−2^	This work
Bi_2.4_Ru_1.6_O_7_	0.1 M KOH	370	[16]
Pb_2_Ru_2_O_6.5_	0.1 M KOH	418	[24]
Sm_2_Ru_2_O_7_	0.1 M KOH	440@2.5 mA cm^–2^	
Y_2_Ru_2-X_Y_X_O_7_	0.1 M KOH	490	[25]
P-Tl_2_Ru_2_O_7_	0.1 M KOH	274	[26]
Bi_2_Ru_2_O_7_	0.1 M KOH	448	[27]
P-Bi_2_Rh_2_O_6.8_	0.1 M KOH	290	[28]
Tl_2_Rh_2_O_7_	0.1 M KOH	423	[29]

## Data Availability

The data presented in this study are available upon request from corresponding author.
